# Human Antimicrobial Peptide LL-37 Inhibits Adhesion of *Candida albicans* by Interacting with Yeast Cell-Wall Carbohydrates

**DOI:** 10.1371/journal.pone.0017755

**Published:** 2011-03-14

**Authors:** Pei-Wen Tsai, Cheng-Yao Yang, Hao-Teng Chang, Chung-Yu Lan

**Affiliations:** 1 Institute of Molecular and Cellular Biology, National Tsing Hua University, Hsinchu, Taiwan; 2 Division of Animal Medicine, Animal Technology Institute Taiwan, Miaoli, Taiwan; 3 Graduate Institute of Molecular Systems Biomedicine, China Medical University, Taichung, Taiwan; 4 Center for Inflammation Research, China Medical University, Taichung, Taiwan; 5 Department of Life Science, National Tsing Hua University, Hsinchu, Taiwan; King's College London Dental Institute, United Kingdom

## Abstract

*Candida albicans* is the major fungal pathogen of humans. Fungal adhesion to host cells is the first step of mucosal infiltration. Antimicrobial peptides play important roles in the initial mucosal defense against *C. albicans* infection. LL-37 is the only member of the human cathelicidin family of antimicrobial peptides and is commonly expressed in various tissues and cells, including epithelial cells of both the oral cavity and urogenital tract. We found that, at sufficiently low concentrations that do not kill the fungus, LL-37 was still able to reduce *C. albicans* infectivity by inhibiting *C. albicans* adhesion to plastic surfaces, oral epidermoid OECM-1 cells, and urinary bladders of female BALB/c mice. Moreover, LL-37-treated *C. albicans* floating cells that did not adhere to the underlying substratum aggregated as a consequence of LL-37 bound to the cell surfaces. According to the results of a competition assay, the inhibitory effects of LL-37 on cell adhesion and aggregation were mediated by its preferential binding to mannan, the main component of the *C. albicans* cell wall, and partially by its ability to bind chitin or glucan, which underlie the mannan layer. Therefore, targeting of cell-wall carbohydrates by LL-37 provides a new strategy to prevent *C. albicans* infection, and LL-37 is a useful, new tool to screen for other *C. albicans* components involved in adhesion.

## Introduction

*Candida albicans* is the major fungal pathogen that affects humans. Although *C. albicans* is a commensal organism on the cutaneous and mucosal surfaces of oral, gastrointestinal, urinary, and vaginal tracts of healthy individuals [1], [2], it is also an opportunistic pathogen and can cause infections ranging from superficial mucosal infections to hematogenously disseminated candidiasis. In immunocompromised patients, *C. albicans* is responsible for a number of life-threatening infections [1], [2]. Moreover, with the rapidly expanding use of medical devices (e.g., indwelling catheters) and increases in the number of patients receiving antibiotic and immunosuppressive therapies, there is an increased risk of fungal penetration through mucosal barriers with subsequent entry into the blood stream, which often leads to multi-organ infections. Consequently, *C. albicans* is the leading cause of nosocomial bloodstream infections and has a mortality rate of 40% [3], [4].

*C. albicans* pathogenesis studies have suggested that several steps may lead to mucosal infection, i.e., early colonization, invasion, and late tissue disruption [5], [6]. *C. albicans* first colonizes and proliferates on the mucosal surfaces of host epithelial cells; these events are followed by invasion and tissue damage [7], [8]. Therefore, *C. albicans* adhesion is the first step in infection and allows the pathogen to persist on mucosal surfaces. As the outermost layer of *C. albicans*, the cell wall interacts with the host cells [9]. The cell wall of *C. albicans* contains many different carbohydrates and proteins that come into contact with epithelial cells and facilitate cell-cell interconnections [10], [11].

Host cells defend against *Candida* infection by producing cytokines [12], chemokines [13], and antimicrobial peptides (AMPs) [14], [15]. Human AMPs are the first line of mucosal immunity [16], [17]. AMPs are generally short (10 to 50 amino acid residues), positively charged (generally +2 to +9), and contain ≥30% hydrophobic residues [18]. Consequently, when folded, many of these peptides exhibit amphiphilic, helical structures and can form pores in microbial membranes, which causes membrane rupture and eventual cell death [19]. Recent studies have indicated that AMPs can also inhibit the biosynthesis of microbial cell walls, nucleic acids, and proteins, and can inhibit the activity of certain microbial enzymes [19], [20], [21], [22]. In humans, different types of AMPs are synthesized and secreted by various cells and tissues, including skin, mucosal surfaces, neutrophils, and epithelia [23].

LL-37 is the only member of the human cathelicidin family of AMPs [24]. LL-37 is stored in specific neutrophil granules as an inactive propeptide, which is cleaved extracellularly to yield the mature, active peptide [25]. In addition to exhibiting broad-spectrum antimicrobial activity against bacteria and fungi, LL-37 has other activities related to host defense, including chemotactic migration, endotoxin neutralization, angiogenesis, and wound healing [26], [27]. To date, studies concerning the effects of LL-37 on *C. albicans* have been few in number. In a study of the candidacidal activity of AMPs, LL-37 was found to remain associated with the *C. albicans* cell surface, whereas other AMPs (e.g., histatin 5) translocated through the membrane and accumulated intracellularly [28].

For the study reported herein, we further investigated the effects of LL-37 on *C. albicans*. We show that LL-37 significantly reduced *C. albicans* attachment to an abiotic surface, to oral epidermis, and to murine urinary bladders. In addition, LL-37 was found to associate with *C. albicans* cell-wall carbohydrates, which caused the cells to aggregate and consequently may help protect host cells against infection. To our knowledge this is the first report showing that LL-37 can interfere with the adhesion of a fungal pathogen to human cells. These results suggest the potential for new therapeutic agents that can target the cell-wall carbohydrates of *C. albicans* and the use of AMPs to prevent *C. albicans* colonization and infection. Moreover, LL-37 might be used to screen for other adhesion molecules on the cell surface of *C. albicans* and/or other fungal pathogens.

## Results

### LL-37 kills *C. albicans*

**Figure 1:**
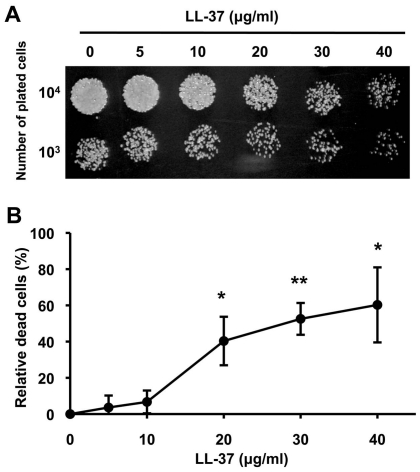
Candidacidal activity of LL-37. (A) Candidacidal activity of LL-37 was determined using a spot assay. The *C. albicans* SC5314 strain was treated with different concentrations of LL-37, and then cells that had been ten-fold serially diluted were spotted onto YPD agar. All experiments were performed in triplicate. (B) The candidacidal activity of LL-37 was monitored by FUN-1 staining. LL-37-treated cells were stained with FUN-1 for 30 min at 30°C. The number of cells killed by LL-37 was normalized with respect to the number of control cells (no LL-37 treatment) and reported as a percentage. Data are presented as the average of three independent experiments, and the statistical significance of the experimental data in comparison to the control data was determined using Student's *t*-test (*, *p*<0.05; **, *p*<0.01).

The ability of LL-37 to kill *C. albicans* was measured by a spot assay and a FUN-1 assay that incorporated flow cytometry (21). For the spot assay, cell viability after LL-37 treatment was visually compared with that of control cells (no LL-37 treatment). We found that cell viability was more sensitive to higher concentrations of LL-37 (20 to 40 µg/ml) as opposed to lower concentrations (5 and 10 µg/ml) or no treatment (Fig. 1A). To quantitatively measure cell mortality, FUN-1 staining was used to distinguish between dead and living cells. Concentrations of LL-37 ≥20 µg/ml induced cell death. At 40 µg/ml LL-37, ∼60% of the cells were killed (Fig. 1B). Therefore, LL-37 had a candidacidal effect on *C. albicans* at doses ≥20 µg/ml.

### LL-37 affects adhesion of *C. albicans* to polystyrene

**Figure 2:**
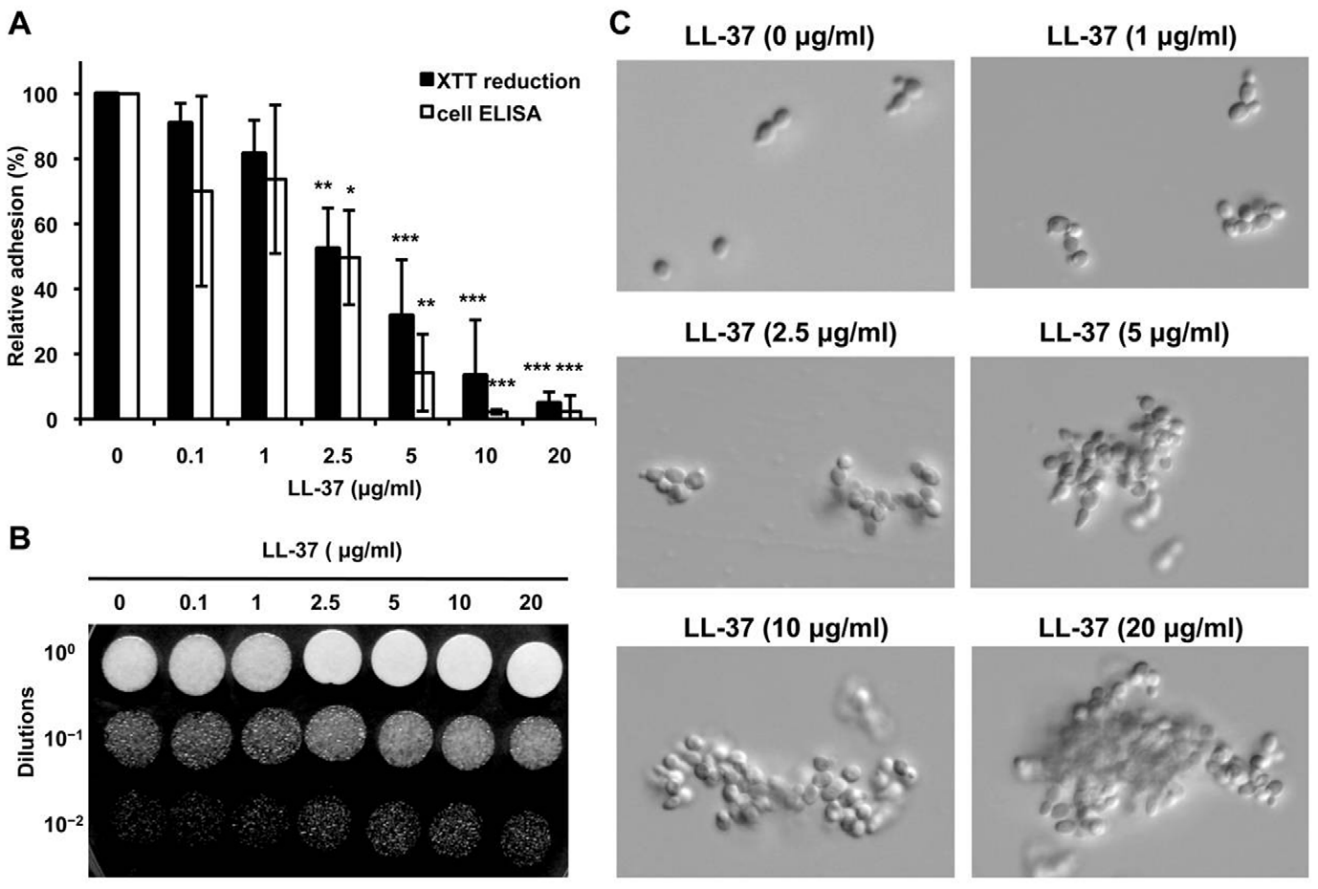
Inhibition of *C. albicans* adhesion by LL-37. (A) Cell adhesion to an abiotic surface (polystyrene). *C. albicans* cells were incubated in RPMI-1640 in polystyrene wells for 30 min and then washed three times with PBS to remove unattached cells. The attached cells were detected by measuring the reduction of XTT and by whole-cell ELISA using an antibody against *C. albicans*. All experiments were done in triplicate, and each was repeated three (XTT reduction assay) or four times (cell ELISA). The statistical significance for the number of treated vs. control cells was determined using Student's *t*-test (*, *p*<0.05; **, *p*<0.01; ***, *p*<0.001). (B) Spot assay to determine the number of viable, floating cells after LL-37 treatment. Different concentrations of LL-37 were incubated with cells in RPMI-1640 for 30 min; then the floating cells were collected, diluted onto YPD agar, and incubated at 30°C overnight. All experiments were done in triplicate, and each was repeated twice. (C) The morphology of the floating cells after LL-37 treatment was visualized by microscopy (400× magnification).

We found that after treatment with 5 µg/ml LL-37, cells were more easily centrifuged and did not adhere to centrifuge tubes to the extent that control cells did (Supplement Fig. S1). Cell adhesion is the first step during *C. albicans* colonization of host tissues and is required for infection. Adhesion is conferred by the binding of *C. albicans* adhesins to amino acid or sugar residues on the host cell surface [29]. Adhesins may also promote cell binding to abiotic substrates, e.g., plastic prostheses and catheters [29]. We therefore suspected that LL-37 might affect interactions between the *C. albicans* cells and the substratum upon which they can proliferate. To test this hypothesis, *C. albicans* were treated with sub-lethal doses of LL-37 (0.1–10 µg/ml), and the extent of *C. albicans* attachment to polystyrene dishes was measured using the 2,3-bis-(2-methoxy-4-nitro-5-sulfophenyl)-2H-tetrazolium-5-carboxanilide (XTT) reduction assay and the whole-cell enzyme-linked immunosorbent assay (ELISA). The extent of attachment decreased with increasing LL-37 concentration (Fig. 2A). Floating cells were collected and spotted onto YPD agar to assess cell viability. The number of colonies derived from these cells depended on the LL-37 concentration and increased between 0.1 and 10 µg/ml LL-37 (Fig. 2B), suggesting that the LL-37 concentrations used in the adhesion assays were not substantially candidacidal. Together, the results shown in Fig. 1 and 2A suggested that low doses of LL-37 inhibited the adhesion of *C. albicans* to polystyrene, that this effect was not directly related to the ability of LL-37 to kill the cells (Fig. 2B), and that mechanisms not involved in the candidacidal effect might be responsible for adhesion loss. We hypothesized that one such mechanism might involve a direct interaction between LL-37 and fungal cells, which was supported by the finding that the fungal cells aggregated after treatment with LL-37 (Fig. 2C). Morphological structures, e.g., the germ tube or hyphae, tend to cause aggregation of *C. albicans* cells. However, as seen in Fig. 2C, most aggregated cells were in the form of yeast. Therefore, LL-37, but not cell morphology, caused the cells to aggregate.

### LL-37 induces cell aggregation and reduces cell attachment to polystyrene by directly binding to *C. albicans*

**Figure 3:**
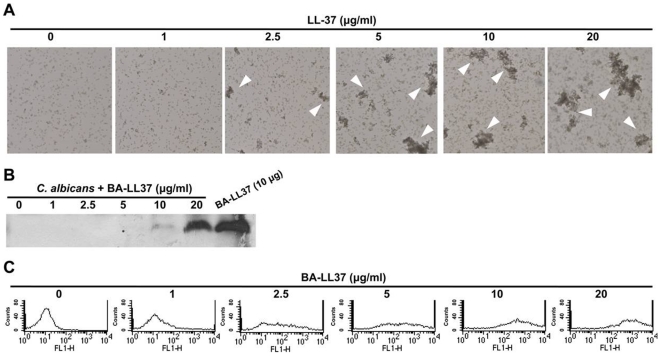
LL-37-induced *C. albicans* cell aggregation and LL-37 binding to *C. albicans*. (A) Visualization by microscopy of *C. albicans* cell aggregation after LL-37 treatment. *C. albicans* cells were incubated with LL-37 in RPMI-1640, and the floating cells were then injected into μ-Slides and visualized by microscopy (250× magnification). Cell aggregates are indicated by the arrowheads. (B) Western blot showing that LL-37 bound to floating *C. albicans* cells. *C. albicans* were incubated with BA-LL37 in RPMI-1640, and the contents of the floating cells were subjected to Tricine SDS-PAGE, blotted, and detected with SA-HRP. BA-LL37 (10 µg) served as the positive control. (C) Flow cytometry showing that LL-37 bound to *C. albicans* cells. Various concentrations of BA-LL37 were incubated with *C. albicans* in PBS at 4°C overnight, and then the levels of cell-bound BA-LL37 were assessed by flow cytometry that used SA-DTAF for detection. The data shown are representative of two additional independent experiments. FL1-H means the extent of fluorescence intensity.

To determine if LL-37 caused the cells to aggregate, which in turn caused them to detach from the plastic dishes, floating cells were collected after LL-37 treatment and characterized by optical microscopy. The extent of floating cell aggregation was directly proportional to LL-37 concentration (Fig. 3A).

Given the dose-dependent effect of LL-37 on cell aggregation, we hypothesized that LL-37 may directly bind to the *C. albicans* cell surface, thereby interfering with cell adhesion to polystyrene. To test this possibility, LL-37-binding assays were performed. *C. albicans* cells were treated with BA-LL37, and floating cells were collected. BA-LL37 bound to these cells as assessed by western blotting that used horseradish peroxidase–conjugated streptavidin (SA-HRP) for detection. Direct binding of LL-37 to the floating cells was obvious when the LL-37 concentrations were 10 and 20 µg/ml (Fig. 3B). However, smaller numbers of floating cells might have been the cause of the non-detectable LL-37 binding at the smaller doses. To test this possibility, we also assessed LL-37 binding using flow cytometry in conjunction with SA-4,6-dichlorotriazinyl aminofluorescein (SA-DTAF) detection. For this system, the binding of BA-LL37 to *C. albicans* cells was observed at a dose of 1 µg/ml (Fig. 3C). These results strongly indicated that LL-37 directly bound to *C. albicans* and induced cell aggregation, which consequently removed the cells from the polystyrene.

**Figure 4:**
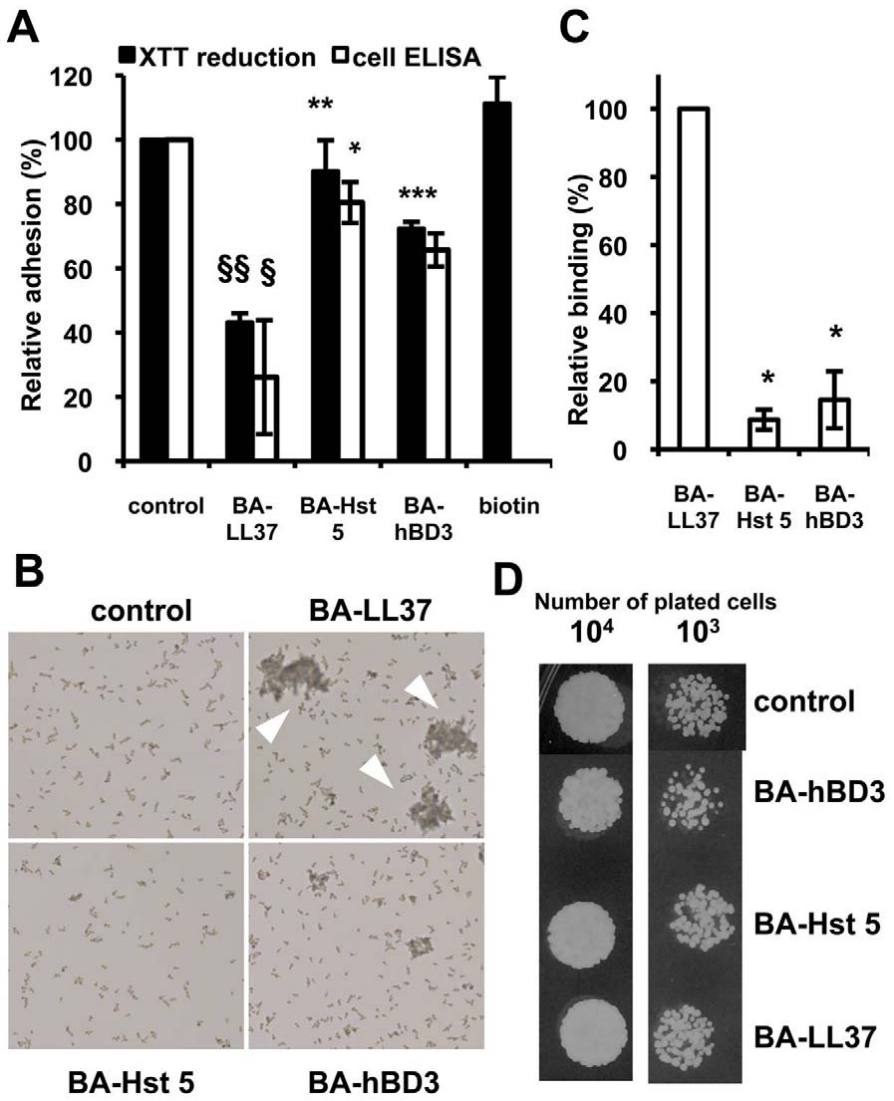
The inhibition of cell adhesion caused by LL-37 is the result of peptide-cell binding and cell-cell aggregation. (A) Effects of BA-LL37, BA-Hst 5, and BA-hBD3 on *C. albicans* adhesion to polystyrene. The molarity of each peptide was 2.12 µM (∼10 µg/ml of BA-LL37). Cells treated with only biotin served as the negative control. This control was performed for only the XTT assay. Each experiment was performed three times in triplicate. The Student's *t*-test was used to determine the statistical significance of the results (§, *p*<0.05 and §§, *p*<0.01 for BA-LL37-treated vs. control cells; *, *p*<0.05; **, *p*<0.01; and ***, *p*<0.001 for BA-LL37-treated cells vs. BA-Hst 5- or BA-hBD3-treated cells as indicated in the figure). (B) Microscopy of *C. albicans* cell aggregation after LL-37 treatment. *C. albicans* cells were incubated in RPMI-1640 with biotinylated AMPs, and the floating cells were collected. Arrowheads indicate BA-LL37-treated cell aggregates. (250× magnification.) (C) Flow cytometry to determine the extent of binding of different AMPs to *C. albicans*. BA-LL37, BA-Hst 5, or BA-hBD3 (10 µg each) were independently added to *C. albicans* samples. The peptide/cell complex was reacted in PBS at 4°C overnight. The Student's *t*-test was used to determine the statistical significance of the results (*, *p*<0.05 BA-LL37-treated cells vs. BA-Hst 5- or BA-hBD3-treated cells). Each result is the mean ± SD of two independent assays. (D) Spot assay to determine the candidacidal activities of the AMPs. BA-LL37, BA-Hst 5, and BA-hBD3 (2.12 µM) were each incubated with *C. albicans* for 30 min in RPMI-1640; then mixtures were diluted, spotted onto YPD agar, and incubated overnight. Each experiment was performed three times.

To investigate if the inhibition of adhesion was LL-37 specific, two other types of AMPs, human β-defensin 3 (hBD-3) and histatin 5 (Hst 5), were tested using the adhesion assay. Hst 5 has a linear, non-helical structure, hBD-3 folds into a β-sheet, and the LL-37 conformation is helical [30]. All three AMPs have candidacidal activity [30]. Equivalent molar concentrations of these AMPs were used in all assays. After *C. albicans* cells were incubated with 2.12 µM BA-hBD3, BA-Hst 5, or BA-LL37 (2.12 µM BA-LL37 is ∼10 µg/ml LL-37), XTT reduction assays and whole-cell ELISAs were performed. In comparison with the level of control-cell adhesion to polystyrene, BA-Hst 5 and BA-hBD3 reduced the adhesion level of *C. albicans* cells by ∼10–30%, whereas BA-LL37 reduced adhesion by 60–80% (Fig. 4A). AMP-induced aggregation of floating cells was then examined by microscopy, and the number of aggregates was recorded. The extent of cell aggregation was greatest for BA-LL37-treated cells, and large aggregates were observed. Conversely, the BA-hBD3-treated cells formed few aggregates, and those treated with BA-Hst 5 did not aggregate (Fig. 4B). Flow cytometry was also used to assess if the AMPs bound to the cells. According to the fluorescence intensities, cells bound by BA-hBD3 and BA-Hst 5 were only 10–15% of that found for BA-LL37-treated cells (Fig. 4C). To exclude the possibility that the inhibitory effects of the AMPs on cell adhesion were caused by cell death, cells were incubated with the same molar concentrations of the AMPs as used for the adhesion assay, and then cell viability was assessed using the spot assay. Cell death was not observed when cells were treated with BA-LL37 or BA-Hst 5, although almost half of the cell population was killed after BA-hBD3 treatment (Fig. 4D). In combination with the results of the cell-adhesion, cell-aggregation, and peptide-cell association assays (Fig. 4), these results indicated that, of the three AMPs tested, only LL-37 inhibited *C. albicans* adhesion to polystyrene by directly binding to cell surface and causing cell aggregation.

### LL-37 binds to *C. albicans* cell-wall polysaccharides and thereby reduces *C. albicans* adhesion

**Figure 5:**
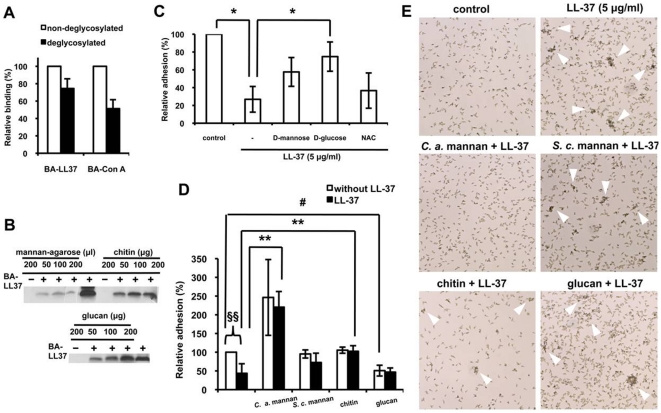
Binding of LL-37 to *Candida* cell-wall polysaccharides reduces *Candida* adhesion to polystyrene. (A) Flow cytometry to determine the relative amount of LL-37 bound to deglycosylated *C. albicans* cells. Cells were mixed with BA-LL37 or BA-Con A, and the binding was measured by SA-DTAF detection. Each result is the mean ± SD of two independent assays. (B) BA-LL37 (10 µg) was incubated with mannan-agarose beads, chitin, or glucan. After washing, the samples were subjected to Tricine SDS-PAGE and detected by western blotting with SA-HRP. BA-LL37 served as the positive control. (C) Monosaccharides reverse the LL-37-mediated inhibition of *C. albicans* cell adhesion. LL-37 (5 µg/ml) was added to *C. albicans* in the absence or presence of 400 µM mannose, d-glucose, and *N*-acetyl-d-glucosamine (NAC), and XTT reduction assays were performed. Each experiment was preformed three times in triplicate. The Student's *t*-test was used to determine the statistical significance of the results (*, *p*<0.05 for LL-37-treated cells in the absence or presence of d-glucose and for LL-37-treated cells vs. control cells). (D) *C. albicans* cell-wall polysaccharides reverse LL-37-mediated inhibition of adhesion. LL-37 (5 µg/ml) was added to *C. albicans* in the absence or presence of 1 mg/ml of a polysaccharide, and then XTT reduction assays were performed. Each experiment was performed three times in triplicate. The Student's *t*-test was used to determine the statistical significance of the results (§§, *p*<0.01 for LL-37-treated cells vs. control cells; #, *p*<0.05 for cells incubated with *S. c.* (*S. cerevisiae*) glucan vs. control cells; **, *p*<0.01 for LL-37-treated cells in the presence of *C. a.* (*C. albicans*) mannan or chitin vs. cells treated only with LL-37). (E) The effects of polysaccharides on the release of LL-37-induced *C. albicans* cell aggregation. Cells were treated as described above. The floating cells were collected and examined under a microscope (250× magnification). Arrowheads indicate aggregates.

The cell wall is the outmost layer of *C. albicans* and, as such, interacts with the environment and host cells [31]. The content of the *C. albicans* cell wall is ∼80–90% carbohydrate and contains different carbohydrates [2]. LL-37 contains an XBBXBX motif (where X is a hydrophobic or uncharged residue, and B is a basic residue) that is responsible for the binding of LL-37 to glycosaminoglycans, e.g., heparin and dermatan sulfate [32]. To evaluate the role of carbohydrates in LL37-mediated inhibition of *C. albicans* adhesion, cell-wall carbohydrates were removed. Concanavalin A (Con A) specifically binds α-d-mannose and α-d-glucose, and BA-Con A was used to monitor the extent of deglycosylation. After deglycosylation, the amount of BA-Con A that bound *C. albicans* cells decreased by ∼50%, which represents the removal of ∼50% of the cell-wall carbohydrates. After removal of the carbohydrates, the binding of BA-LL37 to the *C. albicans* cells was reduced by ∼30% compared with its binding to non-deglycosylated cells (Fig. 5A). Moreover, pull-down assays verified the LL-37/polysaccharide interaction. Since glucan and chitin in solutions will be insoluble, these two polysaccharides and mannan-agarose were used to associate with LL-37 and to pull LL-37 down. Mannan-agarose, glucan, and chitin were each incubated with BA-LL37, after which unbound peptide was separated from the carbohydrates by centrifugation. LL-37 bound directly to these polysaccharides (Fig. 5B). The interaction of each polysaccharide with LL-37 was dose dependent.

To identify the types of carbohydrates targeted by LL-37 binding, the three monosaccharide compositions of the *C. albicans* cell wall, d-glucose, *N*-acetyl-d-glucosamine, and mannose [33], were each included in a cell-adhesion assay. Monosaccharides were pre-incubated with LL-37 to form monosaccharide/LL-37 complex, the inhibition effect of LL-37 on adhesion was thereby suppressed. As shown in Fig. 5C, d-glucose significantly rescued LL37-mediated adhesion inhibition while *N*-acetyl-d-glucosamine and d-mannose also possessed some potential to neutralize the effect of LL-37. We thus concluded that LL-37 might bind to monosaccharides to prevent cell adhesion of *C. albicans* (Fig. 5C). However, polysaccharides, not monosaccharides, account for >90% of the *C. albicans* cell-wall carbohydrates [34], and monosaccharides can not properly reflect structure and function of the cell wall. Therefore, polysaccharides were also tested in adhesion assays (Fig. 5D). In the absence of LL-37, chitin and *Saccharomyces cerevisiae* mannan had no significant effect on cell adhesion, whereas *S. cerevisiae* glucan slightly decreased and *C. albicans* mannan largely increased the level of adhesion the level of adhesion (Fig. 5D). This data was consistent with the finding that *C. albicans* mannan enhances *C. albicans* adhesion to plastic [35]. Moreover, no significant difference was shown by comparing cells with LL-37 and without LL-37 treatment, indicating that LL37-mediated adhesion inhibition of *C. albicans* was abolished in the presence of each polysaccharide. If we considered the effect caused only by LL-37 but not polysaccharedes (i.e. comparing only between the black columns of Fig. 5D), *C. albicans* mannan exhibited the highest extent to relieve LL37-mediated adhesion inhibition, followed by chitin (Fig. 5D). *S. cerevisiae* mannan slightly relieved the adhesion inhibition caused by LL-37 (Fig. 5D). Importantly, the aggregation of floating cells induced by LL-37 was largely reduced by *C. albicans* mannan (Fig. 5E). The aggregations were moderately reduced by chitin but *S. cerevisiae* mannan and glucan had only a minor effect on the floating cell aggregation (Fig. 5E). The dissociation constant (*K*_d_) values for the *C. albicans and S. cerevisiae* mannan/LL-37 complexes were further determined using an Affinity Detection system. The *K*_d_ values were the average of at least two independent experiments. The *K*_d_ for the LL-37/*C. albicans* mannan complex was 0.4±0.1 µM, and that for the LL-37/*S. cerevisiae* mannan complex was 4.9±0.4 µM; this 12-fold decrease in binding strength indicated that LL-37 preferentially binds *C. albicans* mannan. Together, these results demonstrated that LL-37 binds the polysaccharides of the *C. albicans* cell wall, especially mannans, thereby promoting cell aggregation and reducing cell adhesion.

### Effects of LL-37 on *C. albicans* adhesion to oral epidermal cells and the urinary bladders of mice

**Figure 6:**
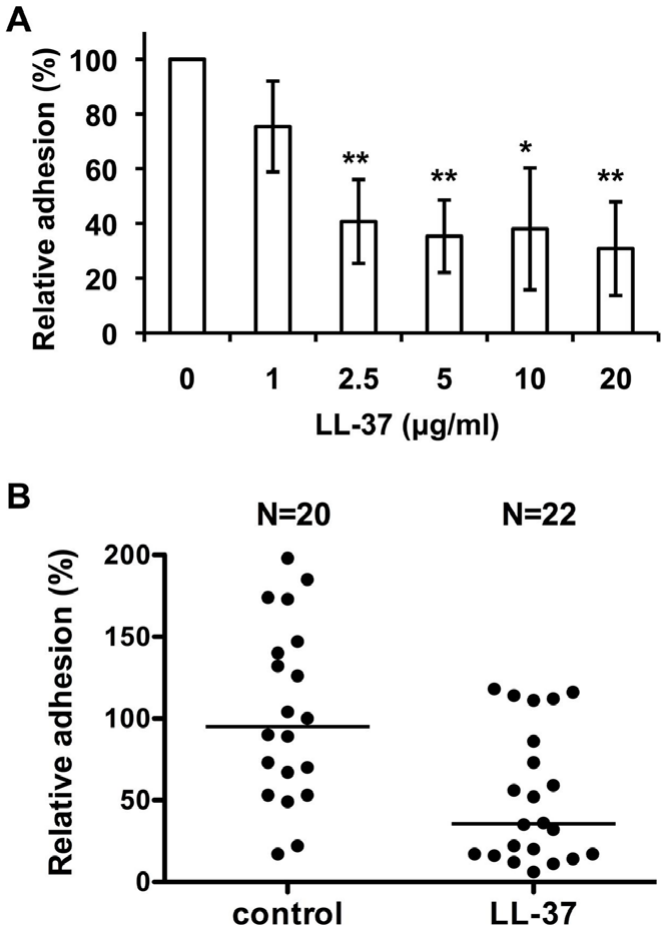
LL-37 inhibits *C. albicans* attachment to oral epidermal cells and mouse bladder mucosa. (A) *C. albicans* cells were incubated in RPMI-1640 at 4°C for 30 min with LL-37, after which the cells were incubated with OECM-1 oral epidermal cells for 30 min. The extent of binding by *C. albicans* was determined by cell ELISA. Each result is expressed as a percentage of that found for untreated cells and is reported as the mean ± SD of four experiments. Each experiment was performed in quadruplicate (*, *p*<0.05; **, *p*<0.01 for LL-37-treated cells vs. control cells). (B) Attachment of *C. albicans* cells to mouse urinary bladders. The number of adhered cells for each bladder was normalized to the average number of cells for all bladders and is reported as a percentage. Three independent experiments were performed with six to eight mice per group. Each horizontal line denotes the median. The difference between 5 µg/ml LL-37-treated cells and controls was significant (*p* = 0.0011; Student's *t*-test).

To assess the *in vivo* effects of LL-37 on *C. albicans* adhesion, we examined the ability of *C. albicans* to adhere to oral epidermal cells and to a murine urinary tract model [36]. *C. albicans* is involved in human oral infections [37], and LL-37 has been shown to diminish the effects of microbial infections in the oral cavity [14]. To determine the extent to which *C. albicans* can adhere to oral epidermal OECM-1 cells, a cell ELISA was performed. After 30 min pre-incubation with LL-37, the cells were added to the OECM-1 cells and incubated for half an hour. The adhesion of the LL37-treated cells was significantly reduced compared with control cells (Fig. 6A). For *C. albicans* treated with 20 µg/ml LL-37, cell adhesion was reduced by >70% (Fig. 6A).

*Candida* species are often isolated from the urine of patients suffering from microbial infection in the bladder or urinary tract [38]. To determine the effects of LL-37 on *C. albicans* adhesion within the urinary tract, we used a mouse model. LL-37 (5 µg/ml) and *C. albicans* were mixed and then injected into the urinary tract. An hour after injection, the number of *C. albicans* cells that had adhered to the bladders was quantified as colony-forming units present on agar. The number of *C. albicans* cells isolated from the bladders when LL-37 was present was significantly less than that of the control (Fig. 6B; *p* = 0.0011). Therefore, LL-37 inhibited *C. albicans* adhesion both *in vitro* and *in vivo*.

## Discussion

Human LL-37 is a multifunctional AMP commonly found at mucosal surfaces at a concentration of ∼2–5 µg/ml [39], [40]. However, its local concentration can be 30–1,500 µg/ml in infected or inflamed tissue [41], [42]. Recently, the effects of LL-37 on bacterial infections have been the focus of much research. In the presence of pathogens, human lung epithelia respond by rapidly increasing LL-37 secretion onto airway surfaces, which subsequently kills nearby microbes [16]. Bacterial contact with urinary tract epithelial cells causes rapid production and secretion of human LL-37 into the urine [36]. Although epithelium-derived LL-37 substantially protects the urinary tract against infection [36], the molecular basis of this host defense mechanism has not been thoroughly characterized.

For the study reported herein, in addition to its known ability to kill *C. albicans*, we demonstrated that human LL-37 can prevent *C. albicans* adhesion to abiotic and biotic substrates. Similarly, LL-37 has been shown to prevent *Escherichia coli* from adhering to the urinary tract of mouse [36] and to inhibit the attachment and biofilm formation of *Pseudomonas aeruginosa* and *Staphylococcus epidermidis* [41], [43] on polystyrene. To our knowledge, however, our report is the first to show that LL-37 blocks the adhesion of a fungal pathogen to substrates. We found that physiological concentrations of LL-37 prevented the adhesion of *C. albicans* to abiotic surfaces *in vitro* and epithelial surfaces *in vivo*. The effects appeared to be specific to LL-37 because Hst 5 and hBD-3, two other candidacidal AMPs, did not inhibit cell adhesion or bind to *C. albicans* to the same degree (Fig. 4A and 4C). LL-37, hBD-3, and Hst 5 have 6, 11, and 12 positive charges, respectively, at physiological pH [30]. In addition, recombinant LL-37 has a propensity to aggregate during purification [44]. Therefore, it is feasible that aggregation of the floating cells was initiated by the direct binding of LL-37 to the cell surfaces and allowed for interaction between LL-37 molecules on adjacent cells (Fig. 4B). At the same molar dose, BA-hBD3 much effectively killed *C. albicans* than BA-LL37 and BA-Hst 5 did (Fig. 4D). Together, these data suggest that the ability of LL-37 to inhibit *C. albicans* adhesion was unrelated to its positive charge or killing activity, but instead was caused by its interaction with the *C. albicans* cell surface, which resulted in cell aggregation and their subsequent inability to bind supporting substrata.

To identify the components of the *C. albicans* cell wall that interact with LL-37, we found that mannan, chitin or glucan and LL-37 associate. The importance of these associations in microbial infection has been reported. Foschiatti and colleagues showed that bacteria use exopolysaccharides to bind and segregate AMPs, thereby reducing the efficiency of the primary innate host defense [45]. Bergsson and colleagues showed that LL-37 binds to glycosaminoglycans from lungs afflicted with cystic fibrosis, which inhibits the ability of LL-37 to kill *Pseudomonas* [46]. In our study, LL-37 association with *C. albicans* cell-wall carbohydrates, in particular mannans, decreased cell adhesion to substrata both *in vivo* and *in vitro*. This ability is crucial for *C. albicans* biofilm formation on an abiotic surface and for colonization on host tissue during infection [10]. Interestingly, LL-37 bound to chitin, glucan, and *S. cerevisiae* mannan, but only *C. albicans* mannan completely rescued LL37-mediated cell aggregation and inhibition of cell adhesion (Fig. 5B, 5D, and 5E). The major difference between the two mannans is that *C. albicans* mannan contains β-1,2 linkages whereas *S. cerevisiae* mannan does not [47]. β-1,2-linked mannans act as the adhesins for *C. albicans*/buccal epithelium complex formation [35], and when the complex was recognized by galectin-3, *C. albicans* death occurred [48]. In an *ex vivo* assay, a monoclonal antibody that recognized β-1,2-mannans inhibited *C. albicans* attachment to murine splenic marginal-zone macrophages [49]. Dromer and colleagues demonstrated that synthetic β-1,2-oligomannosides analogs prevented intestinal colonization by *C. albicans* [50]. Our results indicated that the reduced cell adhesion caused by LL-37 was specifically inhibited by *C. albicans* mannan, suggesting that the association between LL-37 and that particular mannan may involve the latter's β-1,2-linkages. This hypothesis requires further study. Because higher eukaryotic cells do not have a cell wall and because LL-37 exerts its effects by binding to cell-wall components, these components may be drug candidates for *Candida* infection.

Although the polysaccharides tested in our study all interacted with LL-37 (Fig. 5B), when the cells were deglycosylated, BA-LL37 reduced its binding to cell by only ∼30% even though ∼50% of the carbohydrates had been removed (Fig. 5A). Therefore, we cannot exclude the possibility that LL-37 may also interact with other cell-wall components, e.g., proteins. Proteins represent ∼20–30% of the fungal cell-wall mass [11]. Specifically, mannan is covalently bound to proteins (mannoproteins), and this form of mannan accounts for ∼40% of the total cell-wall polysaccharides on the *C. albicans* exterior [51]. Mannoproteins are involved in cell-cell recognition and trigger immune responses [52]. Several studies have showed that AMPs function via their interactions with microbial proteins. The protein, *F. magna* adhesion factor, of the anaerobic bacterium *Finegoldia magna*, blocks the cytocidal activity of LL-37 and promotes *F. magna* colonization and survival in humans [53]. hBD-3 binds to immobilized recombinant hemagglutinin B, a non-fimbrial adhesin from *Porphyromonas gingivalis* [54], [55], which may therefore prevent bacterial adhesion to host tissues [55]. The *C. albicans* proteins possibly targeted by LL-37 and the significance of such LL-37/protein interactions are currently under investigation in our laboratory.

The glucan and chitin layers of the *C. albicans* cell wall are buried beneath a thin but dense mannan layer [11]. We found that LL-37 interacts with mannan, chitin, and glucan (Fig. 5B). Moreover, compared with mannan, chitin and glucan were less able to rescue LL37-mediated cell aggregation and its ability to inhibit cell adhesion (Fig. 5D and 5E). Although this result supports previous findings that mannan is the most important *C. albicans* adhering glycan [56] and that loss of phosphomannan correlates with reduced levels of the AMP dermaseptin S3 bound to the cells [57], it also raises the possibility that LL-37 interferes with *C. albicans* cell-wall remodeling. It is thought that some proteins with enzymatic activity on the *C. albicans* cell surface could be bound by LL-37, leading to dysfunction of carbohydrate metabolism [58], impairment of cell-wall construction, and prevention of *C. albicans*/host adhesion. *C. albicans* cell-wall remodeling occurs during infection and drug (e.g., caspofungin) treatment, and consequently the inner glucan layer is exposed during remodeling [59], [60]. Characterization of the effects caused by LL-37 on cell-wall remodeling will be investigated in future studies.

Finally, AMP-mediated cellular aggregation has been reported. The AMP L8 causes cellular aggregation of *E. coli* and *Listeria monocytogenes*, two important food-borne pathogens [61]. In addition, human neutrophil defensins induce aggregation of *E. coli* and *Staphylococcus aureus* and may, in part, increase bacteria uptake by neutrophils [62]. Similarly, human β-defensins bind bacterial components and thereby enhance microbial ingestion by dendritic cells [63]. We found that in addition to the association of LL-37 and *C. albicans* and the reduction in cell adhesion to polystyrene, floating cells aggregated—mediated by LL-37 bound to the cells (Fig. 2 and 3). Although the biological function of cell aggregation is not clear, we hypothesize that LL37-mediated aggregation may help *C. albicans* escape further attack by LL-37 or may allow phagocytes to easily recognize the pathogen.

In summary, our results revealed that LL-37 prevents *C. albicans* adhesion to polystyrene and tissues, in part by interacting with cell-wall carbohydrates. Our study highlights the potential use of LL-37 as an effective therapeutic or preventive agent against *C. albicans* infection. *C. albicans* polysaccharides could also be targeted by monoclonal antibodies or short peptides to block cell adhesion during infection.

## Materials and Methods

### Peptides and reagents

LL-37 (LLGDFFRKSKEKIGKEFKRIVQRIKDFLRNLVPRTES), biotinylated LL-37 (BA-LL37), biotinylated human β-defensin-3 (BA-hBD3, QKYYCRVRGGRCAVLSCLPKEEQIGKCSTRGRKCCRRKK), and biotinylated histatin 5 (BA-Hst 5, DSHAKRHHGYKRKFHEKHHSHRGY) were synthesized by MDBio, Inc. (Taipei, Taiwan). HPLC and mass spectrometry showed that the peptides were 96–98% pure. All reagents were obtained from Sigma-Aldrich unless indicated otherwise.

### *C. albicans* strains, media, and growth conditions

The *C. albicans* SC5314 [64] cell was maintained at −80°C and plated onto YPD agar (1% yeast extract, 2% Bacto-peptone, 2% glucose and 1.5% agar) before each experiment. One colony was added into YPD broth and incubated at 30°C overnight (∼14 h). This culture was then sub-cultured in YPD broth for ∼2.5 h until logarithmic-phase growth was reached. For LL-37 treatment, cells were washed twice with phosphate-buffered saline (PBS), collected by centrifugation, and suspended in Gibco RPMI-1640 medium (Invitrogen, Carlsbad, CA) or in PBS for LL-37-binding experiments. *C. albicans* cell-wall carbohydrates were removed with GlycoPro™ Enzymatic Deglycosylation kit reagents (Prozyme, San Leandro, CA) according to the manufacturer's instructions.

### Assays for LL-37 candidacidal activity

To assess LL-37 candidacidal activity, a spot assay and the FUN-1 staining assay were used. After a 30-min incubation, LL-37-treated cells were collected and their solutions were subjected to 10-fold serial dilution. Cells (10 µl) were spotted onto YPD agar plates. Cell viability was assessed after incubation at 30°C for 24 h.

FUN-1 staining was performed using LIVE/DEAD® Yeast Viability kit reagents (Molecular Probes, Eugene, OR) according to the manufacturer's instructions and (21). Briefly, after LL-37 treatment for 30 min, *C. albicans* cells were harvested by centrifugation and suspended in 500 µl of 10 mM HEPES containing 2% glucose and 5 µM FUN-1 for 30 min at 30°C in the dark. Samples were then examined using a FACSCalibur analyzer (BD Bioscience, San Jose, CA) equipped with a diode laser (excitation wavelength 488 nm). The intensities of the yellow-green fluorescence emission from cells that had or had not been treated with LL-37 were recorded after the light had passed through an FL1 filter (515–545 nm). A total of 10,000 cells were acquired per experiment. The mean fluorescence index of the LL37-treated cells was compared with that of the control cells (no LL-37 treatment). The relative number of dead cells is reported as a percentage.

### *C. albicans* adhesion to polystyrene

The adhesion of *C. albicans* was assayed using uncoated, flat-bottom, 24-well plates (Orange Scientific, Braine-I'Alleud, Belgium) or uncoated, 96-well plates (Nunc™, Rochester, NY) as described [65], [66]. For the XTT reduction assay, *C. albicans* cells were harvested, washed, and suspended in RPMI-1640 medium at a concentration of ∼6×10^7^ cells/ml. Next, 250 µl of each cell suspension was transferred into a well of a 24-well plate. Different concentrations of LL-37, BA-LL37, BA-hBD3 or BA-Hst 5 were independently added to the cell suspensions that were then incubated at 37°C for 30 min with shaking (100 rpm). The metabolic activities of the sessile cells were measured by detecting the reductive adduct of XTT [67]. Briefly, cells were washed three times with PBS to remove floating cells. Adherent cells were incubated with 300 µl XTT (1 mg/ml) and 0.6 µl menadione (1 µM) in PBS per well at 37°C for 20 min. Absorbance at 490 nm was determined using a VICTOR3 Multilabel Plate Reader (PerkinElmer). For the competition assays, LL-37 was premixed with 400 µM of a monosaccharide or 1 mg/ml of a polysaccharide in RPMI-1640 for 30 min at 4°C before incubating the LL-37/polysaccharide mixture and the cells. All carbohydrate stock solutions were prepared in PBS. *C. albicans* cell-wall mannan was obtained from Takara Bio Inc. (Otsu, Japan). Mannan from *S. cerevisiae* (M7054), glucan from *S. cerevisiae* (G5011), and chitin from crab shells (C9752) were purchased from Sigma-Aldrich.

Cell ELISAs were performed using a similar procedure, except that 100 µl of 5×10^7^ cells/ml were used for each assay. The adherent cells were fixed with 3% paraformaldehyde and labeled with FITC-conjugated rabbit polyclonal antibody against *C. albicans* (Biodesign International, Saco, ME). Fluorescence was detected using a VICTOR3 Multilabel Plate Reader. The relative number of adherent cells was calculated as: [(mean absorbance or fluorescence for each treated cell sample)/(mean absorbance or fluorescence for the control sample)]×100%. All assays were performed in triplicate and repeated three or four times.

### Microscopic visualization of floating *C. albicans* cells

After incubation with different LL-37 concentrations in the wells of 24-well plates, the floating *C. albicans* cells were collected and injected into μ-Slides VI flat (Ibidi, Martinsried, Germany). Samples were analyzed with a Zeiss light microscope.

### Assays for LL-37 binding to floating *C. albicans* cells

*C. albicans* cells were suspended in PBS, mixed with BA-LL37 in the wells of 24-well plates, and incubated at 4°C for 30 min. Floating cells were collected by centrifugation, suspended in reducing sample buffer, and denatured by heating at 100°C for 10 min. The samples were separated through a 15% Tricine SDS-PAGE gel (prepared with a 40% acrylamide/Bis solution; MDBio, Inc., Taipei, Taiwan), and transferred onto a polyvinylidene difluoride (PVDF) membrane (Pall Corporation, Port Washington, NY). BA-LL37 binding to *C. albicans* was detected using SA-HRP according to the manufacturer's instructions (Zymed Laboratories, San Francisco, CA). The blots were processed using ECL kit reagents (PerkinElmer Life Sciences).

### Binding of antimicrobial peptides to *C. albicans* cells

AMPs bound to *C. albicans* were detected as described [48]. A total of 6×10^6^ cells were mixed with different concentrations of BA-LL37 or with 2.12 µM BA-LL37, BA-hBD3, or BA-Hst 5 in 750 µl PBS and then incubated at 4°C overnight. Cell-bound peptide was assessed by flow cytometry with SA-DTAF detection (3 µg/reaction; Jackson ImmunoResearch, West Grove, PA). Reactions were quantitated using a FACSCalibur flow cytometer. Fluorescence data for 10,000 cells were acquired per experiment. The relative amount of an AMP bound to the cells (as a percentage) was obtained by comparing the mean fluorescence intensity emitted from the BA-hBD3- or BA-Hst 5-treated cells with that of BA-LL37-treated cells. Additionally, the relative levels of BA-LL37 and BA-Con A bound to cells that had or had not been deglycosylated were determined.

### Binding of LL-37 to polysaccharides

Polysaccharide binding assays were performed as described [68] with modifications. Mannan-agarose (M9917; Sigma-Aldrich), glucan, and chitin were each incubated with 10 µg BA-LL37 in 750 µl PBS with gentle rotation at 4°C overnight. The samples were pelleted and washed twice with 1 ml PBS. The pellets were suspended in reducing sample buffer and denatured by heating at 100°C for 10 min. The polysaccharide-bound BA-LL37 samples were electrophoresed through a 15% Tricine SDS-PAGE gel, transferred to a PVDF membrane, and detected using SA-HRP and ECL kit reagents according to the manufacturer's instructions.

### Measurement of dissociation constants for LL-37/mannan complexes

Dissociation constants (*K*_d_) for the LL-37/mannan complexes were measured using quartz crystal microbalance methodology [69]. The amine surfaces of gold electrodes on a quartz chip were purchased from ANT Technology Co., Ltd. (Taipei, Taiwan) and were activated with 2.5% glutaraldehyde. Mannans from *S. cerevisiae* and *C. albicans* (100 mg/ml each) were each cross-linked with glutaraldehyde and then coated onto a chip. The binding assay was performed using an Affinity Detection System (ANT Technology). Before each experiment, the system was equilibrated with PBS (pH 7.4) at the flow rate of 50 ml/min. For *K*_d_ measurements, the LL-37 samples (0.1 to 1.6 µM for mannan from *C. albicans*; 1 to 10 µM for mannan from *S. cerevisiae*) were individually injected onto a chip surface that contained one of the yeast cell-wall mannans. All the experiments were performed in duplicate (for mannan from *C. albicans*) or triplicate (for mannan from *S. cerevisiae*). The *K*_d_ values were calculated using Prism 5.0 (GraphPad Software Inc., La Jolla, CA).

### Adhesion of *C. albicans* to oral epidermal cells

The oral epidermoid carcinoma cell line (OECM-1) was kindly provided by Dr. Tzong-Ming Shieh (China Medical University, Taiwan). The cells were grown in RPMI-1640 medium containing 10% (v/v) heat-inactivated fetal bovine serum (BSR BIO), 2 mM l-glutamine, 100 U/ml penicillin, and 100 µg/ml streptomycin (Biosera East Sussex, UK) at 37°C under a 5% CO_2_ atmosphere.

To determine how well *C. albicans* adhered to the oral epithelial cells, a protocol similar to that of the cell ELISA experiment was used, except that 5×10^4^ OECM-1 cells were first grown to 95% confluency in wells of a 96-well plate. Thirty minutes prior to the start of the experiment, 100-µl samples of the cells (1×10^7^ cells/ml) were prepared in RPMI-1640 that contained different concentrations of LL-37 at 4°C. Then, the treated *C. albicans* cells were incubated with the OECM-1 cells at 4°C for another 30 min. The percentage of adhered cells was calculated as follows: [(mean fluorescence for LL-37-treated cells)/(mean fluorescence for control cells)]×100%. All assays were performed in quadruplicate and repeated four times.

### Mouse model for *C. albicans* adhesion

All animal studies were approved by the Institutional Animal Care and Use Committees of the National Tsing Hua University (approval number 09730) and the Animal Technology Institute Taiwan (approval number 97010). Adhesion of *C. albicans* to mouse bladder epithelia was assayed as described [36] with modifications. Briefly, 20- to 24-week-old female BALB/c mice (BioLasco Taiwan Co., Ltd.) were deprived of water overnight. Mice under anesthesia were each transurethrally catheterized with 100 µl of a LL-37 (5 µg/ml) and *C. albicans* mixture (2×10^7^ cells) using soft, sterile polyethylene tubing (PE 10, outside diameter 0.61 mm, inside diameter 0.28 mm, Clay Adams, Becton Dickinson) that had been lubricated. After 1 h of catheterization, the mice were sacrificed and their urinary bladders were removed. The bladders were washed four times with 1 ml PBS and homogenized using a tissue grinder. The number of colony-forming units of *C. albicans* cells that adhered to each bladder was determined by plating serially diluted samples (in PBS) onto YPD agar that contained 50 µg/ml ampicillin and incubating the plates at 30°C overnight. Relative cell adhesion was calculated as follows: [(number of colony-forming units of each bladder/(average number of colony-forming units of all bladders]×100%. Differences in the adhesion for *C. albicans*–injected mouse bladders were determined using GraphPad Prism 5.0 software.

### Statistical analysis

Data were assessed for statistical significance by the two-tailed Student's *t*-test.

## Supporting Information

Figure S1 **Adhesion of LL-37-treated cells to centrifugation tubes.** *C. albicans* was treated with 5 µg/ml LL-37 for 30 min, pelleted by centrifugation, and then the number of cells adhering to the tubes was compared with that of untreated cells by visual inspection. Upper panels: 50-ml centrifuge tubes; lower panels: 1.5-ml microcentrifuge tubes; left panels: cells after centrifugation; right panels: cells after removal of the supernatant; left tube in each panel: control cells (no LL-37 treatment); right tube in each panel: cells treated with 5 µg/ml LL-37.
(TIF)
