# Supplementary figures and images for: Human Antimicrobial Peptide LL-37 Inhibits Adhesion of Candida albicans by Interacting with Yeast Cell-Wall Carbohydrates
Source: PLoS One. 2011 Mar 14;6(3):e17755. doi: 10.1371/journal.pone.0017755 (PMC3056723; doi:10.1371/journal.pone.0017755)

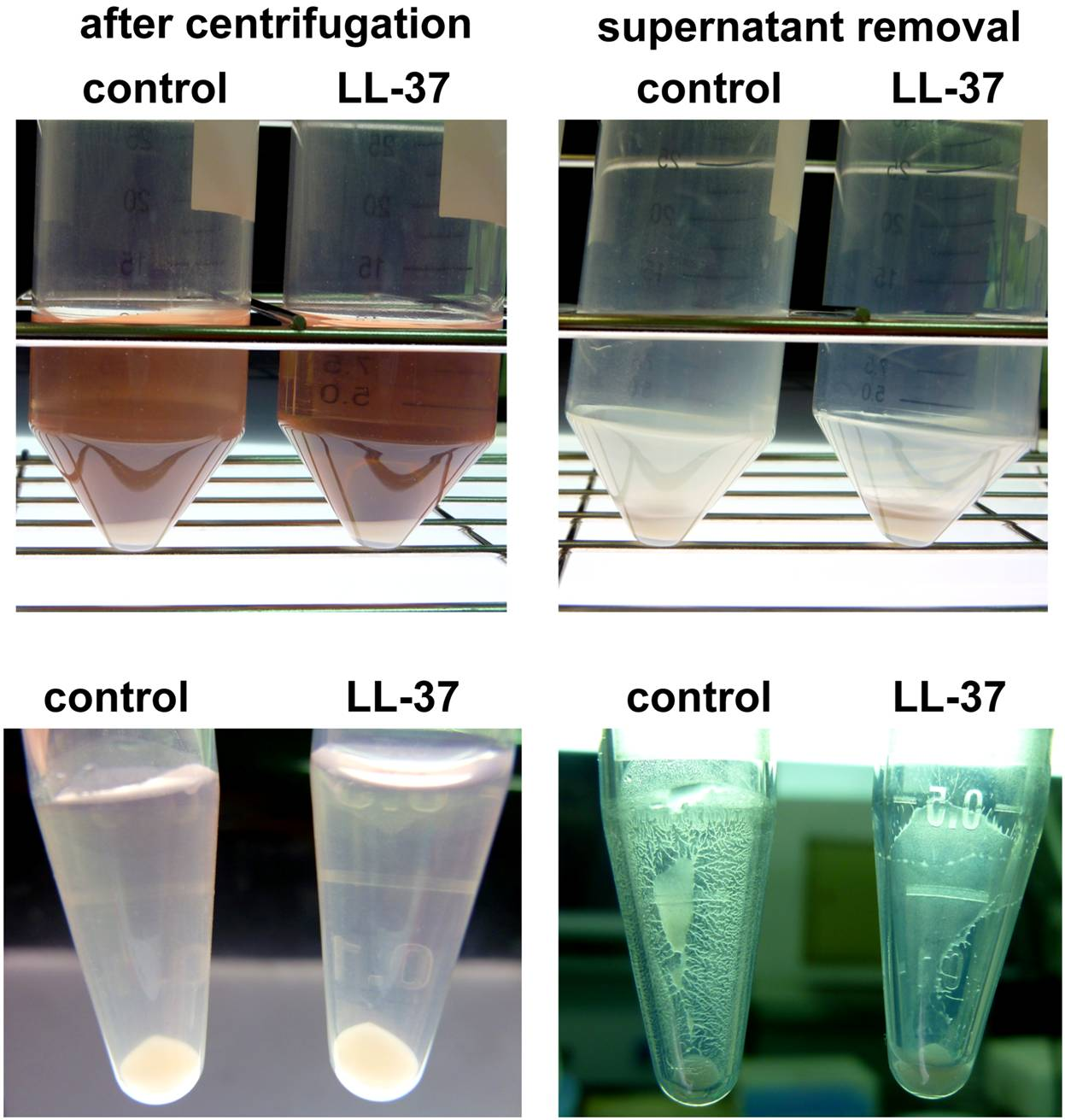

Supplement: Figure S1 — Adhesion of LL-37-treated cells to centrifugation tubes. C. albicans was treated with 5 µg/ml LL-37 for 30 min, pelleted by centrifugation, and then the number of cells adhering to the tubes was compared with that of untreated cells by visual inspection. Upper panels: 50-ml centrifuge tubes; lower panels: 1.5-ml microcentrifuge tubes; left panels: cells after centrifugation; right panels: cells after removal of the supernatant; left tube in each panel: control cells (no LL-37 treatment); right tube in each panel: cells treated with 5 µg/ml LL-37. (TIF) [file pone.0017755.s001.tif]
